# Genetic polymorphisms at *SIRT1* and *FOXO1* are associated with carotid atherosclerosis in the SAPHIR cohort

**DOI:** 10.1186/s12881-014-0112-7

**Published:** 2014-10-02

**Authors:** Lyudmyla Kedenko, Claudia Lamina, Igor Kedenko, Barbara Kollerits, Tobias Kiesslich, Bernhard Iglseder, Florian Kronenberg, Bernhard Paulweber

**Affiliations:** First Department of Internal Medicine, Paracelsus Medical University/Salzburger Landeskliniken, Muellner Hauptstrasse 48, 5020 Salzburg, Austria; Division of Genetic Epidemiology, Innsbruck Medical University, Schöpfstrasse 41, 6020 Innsbruck, Austria; Institute of Physiology and Pathophysiology, Paracelsus Medical University, Strubergasse 21, 5020 Salzburg, Austria; Department of Geriatric Medicine, Paracelsus Medical University/Christian-Doppler-Klinik, Ignaz-Harrer-Strasse 79, 5020 Salzburg, Austria

## Abstract

**Background:**

SIRT1 and FOXO1 interact with each other in multiple pathways regulating aging, metabolism and resistance to oxidative stress and control different pathways involved in atherosclerotic process. It is not known, if genetic polymorphisms (SNPs) at the *SIRT1* and *FOXO1* have an influence on carotid atherosclerosis.

**Methods:**

Intima-media thickness (IMT) was measured on the common and internal carotid arteries. Morphological alterations of the carotid arteries and size of these alterations were included in the B-score grading on a five point scale. Eleven SNPs at *SIRT1* and *FOXO1* gene loci were genotyped in the SAPHIR cohort (n = 1742). The association of each SNP with common carotid IMT, internal carotid IMT and B-score was analyzed using linear regression models.

**Results:**

A significant association was found between common carotid IMT and two SNPs at *FOXO1* - rs10507486, rs2297627 (beta = -0.00168, p = 0.0007 and beta = -0.00144, p = 0.0008 respectively) and at least a trend for rs12413112 at *SIRT1* (beta = 0.00177, p = 0.0157) using an additive model adjusting for age and sex. Additional adjustment for traditional cardiovascular risk factors and markers (BMI, smoking status, hypertension, total cholesterol, HDL-cholesterol, hsCRP) even improved the strength of this association (p = 0.0037 for *SIRT1* and p = 0.0002 for both SNPs at *FOXO1*). Analysis for internal carotis IMT and B-score did not reveal any significant association. One haplotype in *FOXO1* showed a moderate effect on common carotid IMT and B-score in comparison to the reference haplotype of this gene. Several SNPs within *SIRT1* showed differential effects for men and women with higher effect sizes for women: rs3740051 on all three investigated phenotypes (interaction p-value < 0.0069); rs2236319 on common and internal carotid IMT (interaction p-value < 0.0083), rs10823108, rs2273773 on common carotid IMT and rs1467568 on B-score (interaction p-value = 0.0007). The latter was significant in women only (beta_women_ = 0.111, p_women_ = 0.00008; beta_men_ = -0.009, p_men_ = 0.6464).

**Conclusions:**

This study demonstrated associations of genetic variations at the *SIRT1* and *FOXO1* loci with carotid atherosclerosis and highlighted the need for further investigation by functional studies.

## Background

SIRT1 and FOXO1 are evolutionary conserved regulators of aging, metabolic processes and resistance to oxidative stress. Recently, their role in vascular homeostasis has been suggested. SIRT1 seems to have protective properties against atherosclerosis [1]. It controls vascular tone and endothelial function by deacetylation of endothelial nitric oxide synthase and by stimulating its activity [2,3]. In endothelial cells and macrophages SIRT1 has anti-inflammatory functions by down-regulating the expression of various pro-inflammatory cytokines, interfering with the NFκB signaling pathway, preventing macrophage foam cell formation and suppressing of endothelial tissue factor (coagulation factor III) expression [1]. In vascular smooth muscle cells SIRT1 enhances the activity of tissue inhibitor of metalloproteinase 3 that might stabilize atherosclerotic plaques [4]. Moreover, SIRT1 is a key component in several stress-responsive pathways involved in apoptotic cell death and cellular senescence [3].

FOXO1 is strongly expressed in atherosclerotic plaques, regulates expression of different cell cycle regulators and is involved in multiple atherogenic pathways in endothelial cells. FOXO1 activity is regulated through processes of phosphorylation by Akt, acetylation at multiple lysine residues and deacetylation by several deacetylases, including the NAD^+^-dependent deacetylase SIRT1 [5-9].

FOXO1 is deacetylated in response to oxidative stress and hyperglycemia. Deacetylation of FOXO1 leads to its activation and enhanced expression of FOXO1 target genes [10]. Mice bearing constitutively deacetylated alleles of *Foxo1* develop larger artherosclerotic lesions despite improved plasma lipid levels [11]. Ablation of the three genes encoding isoforms of *FOXO* in endothelial cells prevents atherosclerosis in LDL receptor knockout mice [12]. The atheroprotective effect of *FOXO1* deletion was associated with a decrease of insulin-dependent Akt phosphorylation in endothelial cells. Growth factors induce phosphorylation and nuclear exclusion of forkhead proteins in vascular smooth muscle cells that inhibits proliferation of these cells and neointimal hyperplasia [13].

Findings from previous studies showed that SIRT1 and FOXO1 interact with each other in regulating the response to oxidative stress, ageing and longevity. SIRT1 has multiple effects on FOXO-induced gene expression that depresses processes of apoptosis and induces cell-cycle arrest and survival [7,14]. Until now, associations of genetic polymorphisms at *SIRT1* and *FOXO1* with body mass index (BMI), visceral obesity, insulin sensitivity, type 2 diabetes mellitus (T2DM) and all-cause mortality risk have been described [15-19]. Taking into consideration that atherosclerosis is an aspect of the aging process we hypothesized that genetic polymorphisms at *SIRT1* and *FOXO1* may also play a role in carotid atherosclerosis.

## Methods

### Study population

The Salzburg Atherosclerosis Prevention Program in subjects at High Individual Risk (the SAPHIR study) has been initiated in 1999 as a prospective study that investigates genetic and environmental factors contributing to atherosclerotic vascular diseases [20]. Study participants were recruited by health screening programs in large companies in and around the city of Salzburg, Austria. All study participants provided a signed informed consent and the study was approved by the Ethical Committee of Salzburg. All clinical investigations were conducted according to the principles expressed in the Declaration of Helsinki. The study comprises 1770 healthy unrelated Caucasian subjects (663 females and 1107 males aged 39-67 years). At baseline, all participants were subjected to a comprehensive examination – detailed personal and family history, physical, instrumental and laboratory investigations. All laboratory parameters were determined in the fasting state. Participants were diagnosed as having T2DM if their fasting plasma glucose concentration was ≥7 mmol/L and/or they were being treated with anti-diabetic therapy. The participants were placed into hypertension group if the systolic blood pressure was ≥ 140 mm Hg and/or diastolic blood pressure ≥ than 90 mm Hg or they received antihypertensive medication.

### Ultrasonography

Intima-media thickness (IMT) was measured end-diastolic according to the leading edge method for both the near and far walls on the common and internal carotid arteries by high resolution B-mode ultrasound using the ATL HDI 3000 CV (Philips Medical Systems, Bothell, WA, USA) according to the Asymptomatic Carotid Artery Plaque Study (ACAPS) protocol [21]. The protocol included multiple longitudinal and transverse imaging planes of the common carotid artery (CCA) on the 8 mm distance proximal to the bifurcation at height of the flow divider and within the proximal 8 mm of the internal carotid artery (ICA). The mean values of left and right arteries for CCA and ICA were used in the statistical analyses. Because of the technical difficulties in some cases of ultrasonography common carotid IMT and internal carotid IMT were available in 1689 and 1687 participants, respectively. All measurements were conducted and analyzed by a single experienced ultrasound operator who was blinded to all clinical and laboratory data.

The protocol for B-score included multiple longitudinal and transverse imaging planes of the common and internal carotid arteries. Morphological alterations of the carotid arteries and size of these alterations were included in the B-score grading on a 5-point scale: 0 - no alteration, 1 - wall thickness >1 mm, 2 - plaque < 2 mm, 3 - plaque 2 to 3 mm, 4 - plaque > 3 mm, and 5 - total obstruction of the lumen. Adding the B-score of all segments divided by the number of segments resulted in a mean B-score. The mean values of the mean B score were used in the statistical analyses. B-score data were available in 1759 participants.

### Selection of single nucleotide polymorphisms and genotyping

Genotype data of the *SIRT1* and *FOXO1* genes were uploaded from HapMap Data (Phase III/ Rel.#2, Feb 09) (http://hapmap.ncbi.nlm.nih.gov/cgi-perl/gbrowse/hapmap3r2_B36/) and were transferred to SNP tagger (http://www.broad.mit.edu/mpg/tagger/server.html) to identify haplotype-tagging single nucleotide polymorphisms (SNPs). Using the haplotype block structure, we selected a maximally informative subset of validated SNPs with minor allele frequency of >5% for *SIRT1* and >10% for *FOXO1*, which pairwise tag other SNPs within that gene regions with r^2^ > 80%. The difference in selected minor allele frequencies between the *SIRT1* and *FOXO1* genes was based on less polymorphic genetic structure of the *SIRT1* gene. Two SNPs at the *SIRT1* (rs2236319 and rs3740051) were additionally included due to their association with the aging diseases – Alzheimer [22].

Genomic DNA was isolated from whole blood by QIAamp DNA Blood Mini Kit according to manufacture protocol (Qiagen, Germany). DNA samples from whole blood were available for 1742 participants. Genotyping was performed using 5′ nuclease allelic discrimination TaqMan genotyping method and pre-designed assays from Applied Biosystems (Foster City, CA, USA) according to the manufacturer’s instructions.

### Statistical analysis

The association of each SNP was analyzed with log-transformed mean common carotid IMT, mean internal carotid IMT and mean B-score using linear regression models adjusted for a) age and sex and b) age, sex and traditional cardiovascular risk factors and markers (BMI, total cholesterol, HDL-cholesterol, current smoking status, hypertension, hsCRP). For better interpretability of effects, the beta estimates that are reported are based on the outcome-variables on the original scale, whereas p-values are derived from the respective log-transformed models to ensure linear model assumptions. An additive genetic effect was assumed. The analyses were also conducted stratified for sex. In this case analyses were adjusted for age, BMI, total cholesterol, HDL-cholesterol, current smoking status, hypertension, and hsCRP. The occurrence of differential effects between men and women has been evaluated by including SNP*sex interaction terms in the fully adjusted regression model. Additional analyses were performed including pairwise SNP-SNP interaction terms between SNPs of both genes, using the same adjustment models and inheritance assumptions as above.

The Bonferroni correction for multiple testing was applied on an independent number of tests for the main analyses, which are the association analyses of the single SNPs with the three phenotypes. This number was calculated using the effective number of loci [23], which accounts for the correlation structure between the SNPs. Since all three phenotypes are highly correlated, no additional adjustment was made with regard to the number of tested phenotypes or secondary analyses as the interaction analyses. The effective number of loci was estimated to be 6 altogether, three SNPs at both *FOXO1* and *SIRT1.* Therefore, the significance threshold was set to 0.0083 (0.05 divided by 6) for all SNP-based analyses.

Also the haplotypes for *SIRT1* and *FOXO1* genes were estimated by the expectation maximization algorithm using the haplo.stats package (http://CRAN.R-project.org/package=haplo.stats) in the R software environment [24]. Subsequent association analysis of the number of haplotype copies on log-transformed IMT and B-score was adjusted for age and sex. Linkage disequilibrium (LD)-plots were performed using the program Haploview [25]. All other analyses were performed using R software version 2.14.2.

## Results

### Description of the SAPHIR cohort

Clinical and main laboratory characteristics of the SAPHIR cohort are presented in Table 1. Females were older than males and also had higher levels of total cholesterol and HDL-cholesterol in comparison to males.

**Table 1:** **Clinical and laboratory characteristics of the SAPHIR cohort (mean ± SD or n,** %**)**

**Variables**	**All (n = 1770)**	**Males (n = 1107)**	**Females (n = 663)**
**Age, years**	51.39 ± 6.02	48.84 ± 5.42	55.66 ± 4.32
**BMI, kg/m** ^**2**^	26.80 ± 4.12	26.92 ± 3.72	26.58 ± 4.70
**Hypertension, n (%)**	986 (55.7)	590 (53.3)	396 (59.7)
**Smoking, n (%)**	340 (19.2)	254 (22.9)	86 (13)
**T2DM, n (%)**	60 (3.4)	34 (3.1)	26 (3.9)
**Total cholesterol, mg/dl**	228.77 ± 40.13	225.98 ± 39.79	233.46 ± 40.29
**HDL-cholesterol, mg/dl**	59.62 ± 15.72	55.04 ± 13.39	67.30 ± 16.33
**hs-CRP, mg/dl**	0.289 ± 0.625	0.255 ± 0.696	0.347 ± 0.480
**Mean B score**	0.447 ± 0.504	0.421 ± 0.495	0.490 ± 0.518
**CCA IMT, μm**	0.0763 ± 0.0128	0.0758 ± 0.0125	0.0769 ± 0.0131
**ICA IMT, μm**	0.0827 ± 0.0133	0.0827 ± 0.0131	0.0826 ± 0.0135

### Genotype characteristics, linkage disequilibrium and association analysis

**Figure 1:**
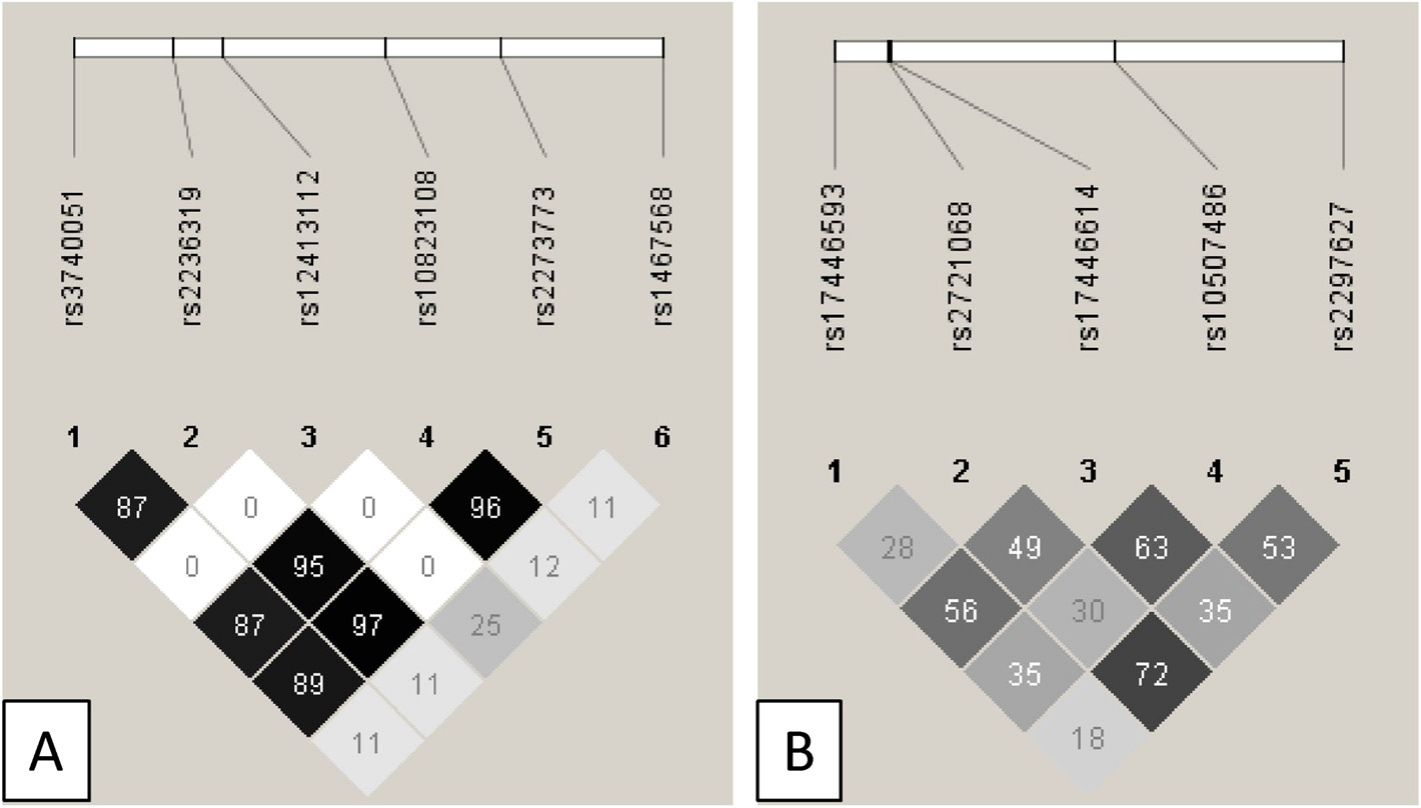
**Linkage disequilibrium structure across the** ***SIRT1*** **(A) and** ***FOXO1*** **(B) single nucleotide polymorphisms.** The pairwise linkage disequilibrium is given for each pair of single nucleotide polymorphisms. Color-coding is based on r^2^.Values denote pairwise r^2^ between single nucleotide polymorphisms.

We genotyped 6 SNPs at *SIRT1* and 5 SNPs at *FOXO1*. Table 2 shows the corresponding descriptive statistics of the genotypes. Two SNPs at *FOXO1* (rs10507486 and rs17446614) showed a deviation from the Hardy-Weinberg equilibrium which was not as extreme to justify exclusion of these SNPs. The pairwise LD in terms of r^2^-values is given in Figure 1. r^2^ is rather high between four SNPs in *SIRT1* (>0.87).

**Table 2:** **Characteristics of the 11 single nucleotide polymorphisms and their genotype quality in the SAPHIR cohort**

	**Alleles**	**SAPHIR (n = 1742)**			
**SNP**	Minor/Major*	**MAF**	Genotype counts**	HWE p***	**Call rate**
***FOXO1***					
rs17446593	G/A	0.178	1186/513/55	1.000	0.991
rs2721068	C/T	0.260	958/666/121	0.755	0.986
rs17446614	A/G	0.155	1259/430/55	0.017	0.985
rs10507486	A/G	0.203	1135/529/91	0.006	0.992
rs2297627	G/A	0.304	867/710/180	0.055	0.993
***SIRT1***					
rs3740051	G/A	0.063	1546/204/8	0.682	0.993
rs2236319	G/A	0.063	1543/207/7	1.000	0.993
rs12413112	A/G	0.120	1365/363/29	0.367	0.993
rs10823108	A/G	0.063	1546/203/9	0.414	0.993
rs2273773	C/T	0.063	1544/205/8	0.683	0.993
rs1467568	A/G	0.340	769/768/209	0.424	0.986

*Minor and Major alleles based on the plus-strand.

**Number of homozygotes for the major allele/heterozygotes/homozygotes for the rare allele.

***Based on exact test of HWE.

Association analysis results for IMT and B-score are presented in Table 3. Significant associations were found between log-transformed mean common carotid IMT and two variants at *FOXO1* (rs10507486, rs2297627) based on an additive model and adjustment for age and sex. These effects are even enhanced after adjustment for traditional cardiovascular risk factors and markers (p = 0.0002). In this multivariate model, a significant effect was also observed for one variant at *SIRT1* (rs12413112, p = 0.0037). The variant allele of rs12413112 at *SIRT1* was associated with an increase of about 0.002 μm in mean common carotid IMT (fully-adjusted model). At the same time variant alleles at the *FOXO1* had an opposite effect and were associated with decreasing IMT (decrease of about 0.0018 μm for rs10507486 or 0.0016 μm for rs2297627).

**Table 3:** **Linear model results on the 11 selected single nucleotide polymorphisms using an additive genetic model**

	**Mean common carotid IMT (n = 1689)**				**Mean internal carotid IMT (n = 1687)**				**Mean B-score (n = 1759)**			
**SNP**	**Age-and sex-adjusted**		**Fully adjusted**		**Age-and sex-adjusted**		**Fully adjusted**		**Age-and sex-adjusted**		**Fully adjusted**	
	**β (se)**	**p-value**	**β (se)**	**p-value**	**β (se)**	**p-value**	**β (se)**	**p-value**	**β (se)**	**p-value**	**β (se)**	**p-value**
***FOXO1***												
rs17446593	-0.00046 (0.00052)	0.3934	-0.00047 (0.00052)	0.3821	0.00041 (0.00057)	0.5654	0.00053 (0.00056)	0.4289	0.00391 (0.02081)	0.7763	0.00648 (0.02066)	0.6889
rs2721068	-0.00089 (0.00045)	0.0412	-0.00106 (0.00045)	0.0145	-0.00049 (0.00049)	0.2739	-0.00055 (0.00048)	0.2224	-0.01029 (0.01803)	0.6473	-0.01329 (0.01786)	0.5229
rs17446614	-0.00118 (0.00054)	0.0272	-0.00125 (0.00053)	0.0180	-0.00032 (0.00058)	0.4625	-0.00027 (0.00058)	0.5278	-0.00860 (0.02131)	0.7509	-0.00725 (0.02118)	0.7789
rs10507486	-0.00168 (0.00042)	**0.0007**	-0.00184 (0.00047)	**0.0002**	-0.00083 (0.00052)	0.1080	-0.00079 (0.00052)	0.1278	-0.02940 (0.01907)	0.1178	-0.03007 (0.01898)	0.1007
rs2297627	-0.00144 (0.00042)	**0.0008**	-0.00158 (0.00042)	**0.0002**	-0.00100 (0.00046)	0.0333	-0.00104 (0.00045)	0.0253	-0.03310 (0.01681)	0.0516	-0.03620 (0.01664)	0.0301
***SIRT1***												
rs3740051	0.00048 (0.00082)	0.5530	0.00053 (0.00081)	0.5012	0.00044 (0.00088)	0.7644	0.00068 (0.00088)	0.5590	0.00096 (0.03260)	0.9972	0.00816 (0.03250)	0.8322
rs2236319	0.00033 (0.00082)	0.7001	0.00039 (0.00081)	0.6382	0.00016 (0.00088)	0.9579	0.00039 (0.00088)	0.7410	-0.00578 (0.03272)	0.8706	0.00484 (0.03254)	0.8668
rs12413112	0.00177 (0.00061)	0.0157	0.00206 (0.00061)	**0.0037**	0.00100 (0.00066)	0.1375	0.00108 (0.00066)	0.1064	0.05019 (0.02423)	0.0847	0.04818 (0.02411)	0.0989
rs10823108	0.00031 (0.00081)	0.6926	0.00038 (0.00080)	0.6102	-0.00002 (0.00087)	0.9096	0.00024 (0.00087)	0.8477	-0.01326 (0.03240)	0.6779	-0.00217 (0.03222)	0.9476
rs2273773	0.00052 (0.00081)	0.5360	0.00059 (0.00081)	0.4716	0.00023 (0.00088)	0.9143	0.00045 (0.00088)	0.7025	-0.00549 (0.03257)	0.8698	0.00502 (0.03239)	0.8692
rs1467568	0.00034 (0.00042)	0.5594	0.00045 (0.00041)	0.3747	0.00063 (0.00045)	0.2329	0.00072 (0.00045)	0.1563	0.03433 (0.01659)	0.0775	0.03577 (0.01645)	0.0643

β-estimate and se: based on original scale of phenotype variables; p-value: based on log-transformed phenotype variables.

Full-adjusted model: age, sex, BMI, total cholesterol, HDL-cholesterol, current smoking status, hypertension, hsCRP.

**In bold**: Significant p-values after correction for multiple testing.

### Haplotype analysis

**Figure 2:**
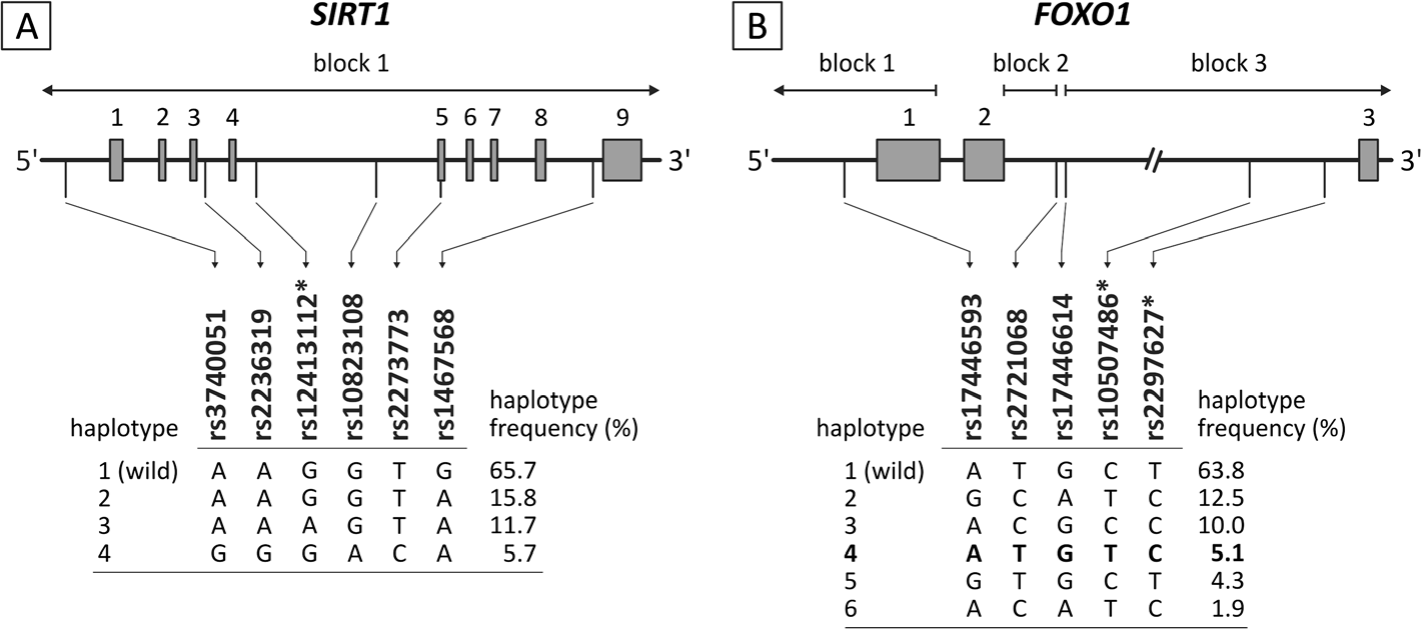
**Genomic structure and haplotypes of the** ***SIRT1*** **and** ***FOXO1*** **genes.** Structure of the *SIRT1* **(A)** and *FOXO1* **(B)** genes; exons are indicated by grey boxes and introns or intragenic regions by horizontal lines. Vertical lines indicate the relative position of the single nucleotide polymorphisms (SNPs) analyzed in this study along with the corresponding haplotypes and haplotype frequencies*.* Haplotype blocks are represented in the upper part. Haplotype which showed association with intima-media thickness (IMT) of the common carotid artery is marked bold. SNP marked with * showed association with common carotid artery IMT.

Only haplotypes with a frequency > 1% were included in the haplotype analysis. Genomic structure of the *SIRT1* and *FOXO1* and their haplotypes are presented in Figure 2.

There was no difference in effects between any haplotypes in *SIRT1* (Table 4*)*. The haplotype ATGTC in *FOXO1* (frequency 5.1%) was associated with CCA IMT and mean B-score in comparison to the reference haplotype (ATGCT) for this gene (β = -0.00326, p = 0.0006 for common carotid IMT and β = -0.09123, p = 0.0077 for B-score). For mean CCA IMT, this haplotype effect primarily reflects the single SNP effects of rs10507486 and rs2297627, since this haplotype differs by alleles at these SNPs from the reference haplotype. For B-score, however, this haplotype analysis detected an association that was not identified on the SNP level.

**Table 4:** **Results of linear regression model for additive haplotype effects (the most common haplotype has been defined as the reference haplotype)**

	**Mean common carotid IMT**		**Mean internal carotid IMT**		**Mean B-score**	
**Haplotype**	**Age-and sex-adjusted**		**Age-and sex-adjusted**		**Age-and sex-adjusted**	
	**β (se)**	**p-value**	**β (se)**	**p-value**	**β (se)**	**p-value**
***FOXO1***						
2	-0.00011 (0.00061)	0.0777	-0.00003 (0.00066)	0.8915	-0.00724 (0.02420)	0.8421
3	-0.00040 (0.00068)	0.4764	-0.00066 (0.00074)	0.3924	-0.01994 (0.02748)	0.5289
4	-0.00326 (0.00089)	**0.0006**	-0.00250 (0.00096)	0.0185	-0.09123 (0.03574)	**0.0077**
5	0.00119 (0.00106)	0.2412	0.00164 (0.00114)	0.1446	0.00922 (0.04323)	0.6623
6	-0.00231 (0.00147)	0.0981	-0.00269 (0.00159)	0.0816	-0.06319 (0.05776)	0.2777
***SIRT1***						
2	-0.00067 (0.00056)	0.2232	0.00040 (0.00060)	0.6033	0.02765 (0.02199)	0.2618
3	0.00163 (0.00063)	0.0363	0.00102 (0.00068)	0.1593	0.05442 (0.02509)	0.0765
4	0.00022 (0.00087)	0.8573	0.00026 (0.00094)	0.9468	-0.00035 (0.03497)	0.8911

β-estimate and se: based on original scale of phenotype variables; p-value: based on log-transformed phenotype variables.

All models are age-and sex-adjusted.

**In bold**: Significant p-values after correction for multiple testing.

### Sex-stratified analyses

SNP*sex interaction terms and sex-stratified analyses revealed additional information on the association of the investigated SNPs with atherosclerosis in the carotid arteries. Differential effects for men and women were observed for several SNPs within *SIRT1* (Table 5): for rs3740051 on all three investigated phenotypes (p interaction <0.0069); for rs2236319 on CCA IMT and ICA IMT (p interaction <0.0083), for rs10823108 and rs2273773 on common carotid IMT only and for rs1467568 on B-score only (p interaction = 0.0007). For all of these SNPs, effect directions differed between men and women with decreasing IMT and/or B-score for each minor allele for men, and increasing IMT and/or B-score for the minor allele for women. In men, these decreasing effects were not significant. In women, all of these differentially effective SNPs were at least nominally significantly associated with their respective phenotypes (all women-specific p-values < 0.013) with about 3-4 times higher effect sizes for women than for men. The highest difference was observed for the rs1467568 at *SIRT1* on the B-score with a significant effect in women only (β_women_ = 0.111, p_women_ = 0.00008; β_men_ = -0.009, p_men_ = 0.6464).

**Table 5:** **Sex-specific effects for the genetic polymorphisms in** ***FOXO1*** **and** ***SIRT1*** **genes, fully adjusted model**

	**CCA IMT (n = 1689)**			**ICA IMT (n = 1687)**			**B score carotis (n = 1759)**		
**SNP**	**β (se) men**	**β (se) women**	**p-value interaction**	**β (se) men**	**β (se) women**	**p-value interaction**	**β (se) men**	**β (se) women**	**p-value interaction**
***FOXO1***									
rs17446593	-0.00139 (0.00062)	0.00114 (0.00091)	0.0147	-0.00005 (0.00068)	0.00149 (0.00097)	0.2658	-0.01506 (0.02505)	0.04379 (0.03610)	0.2623
rs2721068	-0.00125 (0.00055)	-0.00093 (0.00076)	0.4421	-0.00077 (0.00060)	-0.00035 (0.00081)	0.5616	-0.01602 (0.02580)	-0.00667 (0.03023)	0.7809
rs17446614	-0.00164 (0.00063)	-0.00057 (0.00094)	0.2277	-0.00052 (0.00070)	0.00017 (0.00100)	0.5361	-0.01602 (0.02580)	0.00762 (0.03688)	0.7200
rs10507486	-0.00218 (0.00057)	-0.00138 (0.00082)	0.3032	-0.00098 (0.00063)	-0.00058 (0.00088)	0.7306	-0.04115 (0.02326)	-0.01506 (0.03266)	0.6520
rs2297627	-0.00151 (0.00051)	-0.00187 (0.00071)	0.9872	-0.00105 (0.00056)	-0.00120 (0.00076)	0.9945	-0.03709 (0.02058)	-0.03876 (0.02833)	0.9046
***SIRT1***									
rs3740051	-0.00122 (0.00094)	0.00433 (0.00152)	**0.0019**	-0.00104 (0.00104)	0.00458 (0.00162)	**0.0024**	-0.04678 (0.03822)	0.133248 (0.060434)	**0.0069**
rs2236319	-0.00133 (0.00094)	0.00408 (0.00152)	**0.0025**	-0.00108 (0.00103)	0.00372 (0.00162)	**0.0083**	-0.04248 (0.03819)	0.1120283 (0.0606840)	0.0147
rs12413112	0.00140 (0.00073)	0.00309 (0.00106)	0.2166	0.00129 (0.00080)	0.00066 (0.00113)	0.7856	0.02879 (0.02938)	0.0809768 (0.0421386)	0.3940
rs10823108	-0.00113 (0.00092)	0.00383 (0.00153)	**0.0063**	-0.00118 (0.00101)	0.00359 (0.00164)	0.0101	-0.04684 (0.03751)	0.104012 (0.061163)	0.0206
rs2273773	-0.00107 (0.00093)	0.00426 (0.00153)	**0.0030**	-0.00097 (0.00102)	0.00378 (0.00163)	0.0091	-0.04149 (0.03782)	0.11381 (0.06106)	0.0152
rs1467568	-0.00027 (0.00050)	0.00160 (0.00070)	0.0329	-0.00021 (0.00056)	0.00215 (0.00075)	0.0124	-0.00931 (0.02029)	0.110641 (0.027819)	**0.0007**

β-estimate and se: based on original scale of phenotype variables; p-value: p-value from the SNP*gender interaction term of the full-adjusted model, based on log-transformed phenotype variables.

Full-adjusted model: age, sex, BMI, total cholesterol, HDL-cholesterol, current smoking status, hypertension, hsCRP.

**In bold**: Significant p-values after correction for multiple testing.

There were no differential effects between men and women in any of the *FOXO1* SNPs.

## Discussion

Our study is the first to report a significant association of genetic polymorphisms at *SIRT1* and *FOXO1* with a carotid atherosclerosis. More pronounced effects were present for two SNPs (rs10507486 and rs2297627) at *FOXO1* and common carotid IMT. Also we found one haplotype in *FOXO1* with a moderate effect on common carotid IMT and B-score in comparison to the reference haplotype. Finally, we detected a sex-specific effect for the SNPs at *SIRT1* with a protective role of the minor alleles in males and an opposite effect in females.

The pathophysiological functions of SIRT1 and FOXO1 in vascular homeostasis and carotid atherosclerosis have been described within the last years and exert protective roles in case of SIRT1 and controversial roles for FOXO1. Also, the interaction between SIRT1 and FOXO1 in different organisms has been demonstrated. SIRT1 deacetylates FOXO1 and regulates its transcriptional activity, in turn, FOXO1 exerts a positive feedback mechanism regulating *SIRT1* expression. FOXO1 can directly bind to *SIRT1* promoter region and increase *SIRT1* transcription and expression [26]. Phosphorylation of FOXO1 makes it retention in the cytoplasm and inhibition of its transcription activity [27,28]. In response to oxidative stress, FOXO1 translocates to the nucleus, interacts with SIRT1 resulting in the deacetylation of FOXO1 [10]. This depresses the sensitivity of FOXO1 to insulin-induced phosphorylation, resulting in gain function [29]. At the same time, acetylation of FOXO1 facilitates their phosphorylation and nuclear translocation.

Oxidative stress or hyperglycemia induce FOXO1 deacetylation in vascular endothelial cells, that contributes to the increased risk of atherosclerosis in diabetic patients. Experiments with isolated vascular endothelial cells from mice indicated that deacetylated FOXO1 affects its gene expression response to a variety of pathogenic stimuli and increases interaction of monocytes with endothelium [11].

Previous publications have shown an association of genetic variations at *SIRT1* with BMI and visceral obesity [16], acute insulin secretion [30], basal energy expenditure and the respiratory quotient [31]. Carriers of the mutant allele were resistant against lifestyle-induced improvement of fasting plasma glucose, had less increase in insulin sensitivity and an attenuated decline in liver fat [31]. Also it was shown that expression of the *SIRT1* gene was negatively correlated with carotid IMT [32]. However, no genetic polymorphisms in the *SIRT1* gene were associated with expression level of the *SIRT1* transcript [16].

There are also findings about the role of genetic variations in the *FOXO1* and the susceptibility for T2DM. Müssig et al. showed the nominally significant association of rs2297627 with β-cell dysfunction (p = 0.0387) and impaired glucose tolerance (p = 0.0221). Carries of the minor allele had reduced insulin secretion and elevated glucose levels during an oral glucose tolerance test [17]. Kuningas et al. revealed the role of different haplotypes in the human *FOXO1a* gene in glucose metabolism, all-cause and T2DM mortality risk [33].

Association of genetic polymorphisms at the *SIRT1* and *FOXO1* loci with carotid atherosclerosis have not been reported before. However, significant associations between genetic variants in other *SIRT* gene - the *SIRT6* and atherosclerotic plaque have been observed [34]. The last meta-analysis of genome-wide association studies by the CHARGE consortium identified some variants associated with common carotid IMT and plaques. However, no SNP achieved significant thresholds on a genome-wide scale for internal carotid IMT [35].

The genetic effect of *SIRT1* and *FOXO1* on common carotid IMT shown in the current study might be explained by their direct involvement in the pathophysiological mechanisms of atherosclerosis. FOXO1 integrates diverse cellular signals through its post-translational modifications, such as phosphorylation, acetylation, deacetylation resulting in changes in DNA binding properties, transcriptional activity, protein stability and subcellular localization. At the same time, SIRT1 is one of multiple regulators of FOXO1 activity in endothelial and smooth muscle cells.

It has to be noted that the definition of the carotid atherosclerotic phenotype consists of site-dependent IMT measurement and plaque-score. Although IMT and plaques are highly correlated with each other [36,37], we found in our study an association of genetic polymorphisms at the *SIRT1* and *FOXO1* only with common carotid IMT. A haplotype specific effect was also found for mean B-score, though, which could not be detected at the single SNP level. The site-specific differences in atherosclerosis phenotype might be based on the important role of blood flow and genetics in manifestation and progression of carotid atherosclerosis [38-40]. Elevated carotid IMT may be the result of an increased intima layer as a consequence of atherosclerotic process, but also as an increased media layer due to vascular hypertrophy in hypertension or increasing of both layers. Nevertheless, IMT in carotid arteries is an established marker of the early onset of atherosclerosis and has been included in recent guidelines as a potentially useful marker for cardiovascular disease prediction in clinical and epidemiological studies [41,42].

It is evident, that there is a sex-specific aspect of cardiovascular disease. In the Tromso Study, carotid atherosclerosis was a stronger predictor of myocardial infarction in women than in men [43]. Also this study showed that only in women triglyceride levels were associated with an increase in IMT, while physical activity and smoking were predictors of IMT in men [44]. Previously we published that the metabolic syndrome is a stronger risk factor for early carotid atherosclerosis in women than in men. In females, blood glucose and triglyceride levels showed the strongest association with IMT, whereas HDL-cholesterol had a stronger effect in men [45]. The available literature describes sex-specific effects only for *SIRT1* in relation to increased risk for T2DM in Pima Indians [30] and *SIRT6* for atherosclerotic plaque [34]. We cannot explain the mechanism underlying the sex-related association shown in our study. The SNPs that showed sex-specific effects were not significant in the overall analysis. Different effect of genetic polymorphisms in the *SIRT1* on carotid atherosclerosis in female and male populations might be partial explained by the different role of sex hormones in regulation of gene expression or important role of sex-specific risk factors for atherosclerosis.

The strength of this investigation is that all ultrasound investigations were done by a single experienced ultrasound operator who was blinded to all clinical and laboratory measurements. It allowed us to reduce inter-observer variability and obtain data independent from the clinical phenotype. Moreover, the gene targeting approach used in this study gave not only the possibility to find association of genetic variations with carotid IMT and B-score, but also brought a new information about role of genetics of *SIRT1* and *FOXO1* in the atherosclerotic process. There are also some limitations of this investigation. First, replication of the findings in an independent cohort is missing. Due to power issues it was also not possible to perform an internal validation approach. Secondly, some of the included SNPs were in linkage disequilibrium although SNPs have been selected using SNPtagger to exclude such a situation. In the analysis, the LD structure was accounted for by calculating the number of independent SNPs. Therefore, further replication studies and also functional studies are warranted to elucidate the molecular mechanism of this association and the role of these genetic variations at *SIRT1* and *FOXO1* in carotid atherosclerosis.

## Conclusions

Atherosclerosis is a clinically latent process for many years before its manifestation as coronary, cerebrovascular and/or peripheral arteries diseases. The identification of patients with high cardiovascular risk in subclinical phase of atherosclerotic process is critical for primary prevention of cardiovascular diseases. Genetics in combination with clinical parameters and carotid phenotypes may help us to understand pathogenesis of atherosclerotic diseases which might improve the prediction of cardiovascular diseases. However, the functional consequences of *SIRT1* and *FOXO1* genetics in regulation of carotid atherosclerosis remain incompletely understood and should be investigated in further functional studies.

## Abbreviations

BMI: Body mass index
CCA: Common carotid artery
DNA: Deoxyribonucleic acid
FOXO1: Forkhead box protein O1 (protein)
*FOXO1*: Forkhead box protein O1 (gene, human)
*Foxo1*: Forkhead box protein O1 (gene, mouse)
HDL: High-density lipoprotein
hsCRP: High-sensitivity C-reactive protein
HWE: Hardy-Weinberg equilibrium
ICA: Internal carotid artery
IMT: Intima-media thickness
LD: Linkage disequilibrium
LDL: Low-density lipoprotein
MAF: Minor allele frequency
NAD^+^: Nicotinamide adenine dinucleotide
NFkB: Nuclear factor ‘kappa-light-chain-enhancer’ of activated B-cells
SAPHIR: Salzburg Atherosclerosis Prevention Program in subjects at High Individual Risk
SIRT1: Sirtuin 1 (protein)
*SIRT1*: Sirtuin 1 (gene, human)
SNP: Single nucleotide polymorphism
T2DM: Type 2 diabetes mellitus
